# Cost-effectiveness of an adjuvanted recombinant zoster vaccine in older adults in the United States who have been previously vaccinated with zoster vaccine live

**DOI:** 10.1080/21645515.2018.1558689

**Published:** 2019-02-20

**Authors:** Desmond Curran, Brandon J. Patterson, Desiree Van Oorschot, Philip O. Buck, Justin Carrico, Katherine A. Hicks, Bruce Lee, Barbara P. Yawn

**Affiliations:** a Value Evidence, GSK, Wavre, Belgium; b US Health Outcomes & Epidemiology, GSK, Philadelphia, PA, USA; c Health Economics, RTI Health Solutions, Durham, NC, USA; d Global Obesity Prevention Center, Johns-Hopkins University, Baltimore, MD, USA; e Department of Family and Community Health, University of Minnesota, Minnesota, MN, USA

**Keywords:** Herpes zoster, vaccination, cost-effectiveness, revaccination, recombinant zoster vaccine, booster, older adults

## Abstract

Zoster Vaccine Live (ZVL) is marketed in the US since 2008, and a non-live adjuvanted Recombinant Zoster Vaccine (RZV) was approved in 2017. Literature suggests that waning of ZVL efficacy may necessitate additional vaccination. The Advisory Committee on Immunization Practices recommended vaccination with RZV in immunocompetent adults aged 50+ years old, including those previously vaccinated with ZVL. The objective of this study was to determine the cost-effectiveness of vaccinating US adults aged 60+ years old, previously vaccinated with ZVL. The ZOster ecoNomic Analysis (ZONA) model, a deterministic Markov model, was adapted to follow a hypothetical 1 million(M)-person cohort of US adults previously vaccinated with ZVL. Model inputs included demographics, epidemiology, vaccine characteristics, utilities and costs. Costs and quality-adjusted life-years (QALYs) were presented over the lifetimes of the cohort from the year of additional vaccination, discounted 3% annually. The model estimated that, vaccination with RZV 5 years after previous vaccination with ZVL, would reduce disease burden compared with no additional vaccination, resulting in a gain of 1,633 QALYs at a total societal cost of $96M (incremental cost-effectiveness ratio: $58,793/QALY saved). Compared with revaccinating with ZVL, vaccination with RZV would result in a gain of 1,187 QALYs and societal cost savings of almost $84M. Sensitivity, scenario, and threshold analyses demonstrated robustness of these findings. Vaccination with RZV is predicted to be cost-effective relative to no additional vaccination, assuming a threshold of $100,000/QALY, and cost-saving relative to ZVL revaccination of US adults aged 60+ years old who have been previously vaccinated with ZVL.

## Introduction

Herpes zoster (HZ, shingles) is a viral infection elicited by the reactivation of varicella-zoster virus (VZV, chickenpox).^1^

Both the incidence and severity of HZ increases in people aged 50+ years old associated with an age-related decline in VZV-specific cellular immunity.^2^ HZ is a painful and costly condition estimated to result in approximately $1.3 billion in medical care costs and $1.7 billion in indirect costs in the US annually;^3^ this burden is projected to rise substantially over the coming years due to aging populations.^4^

In 2008, the Advisory Committee on Immunization Practices (ACIP) recommended that adults aged 60+ years old be vaccinated against HZ.^5^ A live attenuated zoster vaccine, *Zostavax* (Zoster Vaccine Live [ZVL]) (currently licensed for use in healthy adults aged 50+ years old), is a one-dose vaccine that utilizes the same Oka strain as in varicella vaccines but at a higher potency.^6^ ZVL efficacy shows an inverse relationship with age, with demonstrated efficacy against HZ of 51% in people aged 60+ years old, 38% in people aged 70+ years old and 18% in adults aged 80+ years old.^1,6^ In adults aged 60+ years old, vaccine efficacy of ZVL against HZ declined to 0% by 11 years post vaccination,^7^ suggesting subjects would require additional vaccination to retain protection against HZ.

A recent mathematical modelling study concluded that ZVL revaccination of US adults previously vaccinated with ZVL would be cost-effective after 5 years compared with no additional vaccination and most economically favorable after 10 years.^8^ At the time of the study, non-live adjuvanted Recombinant Zoster Vaccine (RZV) was not available.

*Shingrix* (RZV) was developed to prevent HZ and its complications.^9^ RZV combines glycoprotein E (gE), an abundant VZV surface protein, with an adjuvant system AS01_B_.^10^ RZV demonstrated efficacy results against HZ of 97.2% in subjects aged 50+ years old and 91.3% in subjects aged 70+ years old, remaining at 93.1% and 87.9% respectively 4 years post vaccination.^10,11^ Several studies with subjects who have not been previously vaccinated against HZ have demonstrated that RZV is cost-effective compared with a no vaccination strategy and cost-saving compared with ZVL vaccination.^12–14^

Although there are no head-to-head studies comparing ZVL with RZV, a recent network meta-analyses of clinical trial data demonstrated that vaccine efficacy against HZ was significantly higher using RZV compared with ZVL in adults aged 60+ years old.^15^ Furthermore, it was demonstrated that RZV induces a strong immune response in subjects previously vaccinated with ZVL.^16^ ACIP conducted a review of clinical efficacy data, health economic evidence and immunogenicity data, and in October 2017 recommended RZV 1) in immunocompetent adults aged 50+ years old, 2) in immunocompetent adults previously vaccinated with ZVL, and 3) preferentially over ZVL.^17^

The current study was performed to address the primary research question: “Is RZV cost-effective in US adults aged 60+ years old previously vaccinated against HZ with ZVL?” The cost-effectiveness analysis was carried out comparing RZV vaccination versus no additional vaccination, and versus ZVL revaccination.

## Results

Table 1 presents the results of the base cost-effectiveness analysis. Vaccination with RZV is compared with no additional vaccination and ZVL revaccination of a cohort of 1 million US adults aged 60+ years old previously vaccinated with ZVL. The model estimated that, compared with no additional vaccination, RZV vaccination would prevent 82,769 HZ cases, 8,402 postherpetic neuralgia (PHN) cases, 11,946 other complications, and 14 HZ-related deaths over the remaining lifetimes of all individuals in the model cohort. This reduced disease burden would result in a gain of 68 discounted life-years and 1,633 discounted quality-adjusted life-years (QALYs). The vaccination costs would total $304 million dollars, but the HZ cases prevented would save $163 million in direct costs and $45 million in indirect costs, resulting in a net total societal cost of vaccinating a cohort of 1 million adults of approximately $96 million. These outcomes equate to a net cost of $58,793 per QALY gained. Compared with ZVL revaccination, the model estimated that RZV vaccination would prevent 67,441 additional HZ cases, 6,137 PHN cases, 9,938 other complications, and 13 HZ-related deaths. This reduced disease burden would result in a gain of 59 discounted life-years and 1,187 discounted QALYs. The incremental vaccination costs would total almost $78 million dollars, but the HZ cases prevented would save $129 million in direct costs and over $33 million in indirect costs, resulting in net total societal cost savings of approximately $84 million.

**Table 1.** Base-case analysis results for 1 million of US adults aged 60+ years old previously vaccinated with ZVL, comparing no additional vaccination, vaccination with RZV, and ZVL revaccination.

Outcome	No additional vaccination	Vaccination with RZV	ZVL revaccination	RZV vs no additional vaccination	RZV vs ZVL revaccination
**Health Outcomes**					
HZ cases	176,801	94,033	161,474	(82,769)	(67,441)
PHN cases	20,173	11,771	17,908	(8,402)	(6,137)
Other Complication cases	27,098	15,152	25,090	(11,946)	(9,938)
Ocular	9,931	5,630	9,212	(4,301)	(3,582)
Neurological	8,926	4,807	8,203	(4,119)	(3,396)
Cutaneous	4,078	2,348	3,806	(1,730)	(1,458)
Other non-pain	4,163	2,368	3,870	(1,796)	(1,502)
HZ-related deaths	47	34	46	(14)	(13)
**Costs (discounted)**					
Vaccination costs	–	$304,405,178	$226,897,269	$304,405,178	$77,507,909
Direct costs due to HZ	$325,979,303	$162,986,740	$291,629,995	($162,992,563)	($128,643,255)
Indirect costs due to HZ	$72,960,889	$27,579,322	$60,934,650	($45,381,567)	($33,355,328)
Total direct costs	$325,979,303	$467,391,918	$518,527,264	$141,412,615	($51,135,346)
Total societal costs	$398,940,193	$494,971,240	$579,461,914	$96,031,047	($84,490,674)
**Life-years/QALYs (discounted)**					
Life-years	12,890,621	12,890,689	12,890,630	68	59
QALYs	10,120,248	10,121,881	10,120,694	1,633	1,187
**Cost-effectiveness**					
Incremental cost per QALY gained	–	–	–	$58,793	Cost saving

–: not applicable; () refers to savings. HZ: herpes zoster; PHN: postherpetic neuralgia; QALY: quality-adjusted life-year; RZV: adjuvanted recombinant zoster vaccine; US: United States; ZVL: zoster vaccine live.

**Figure 1.**
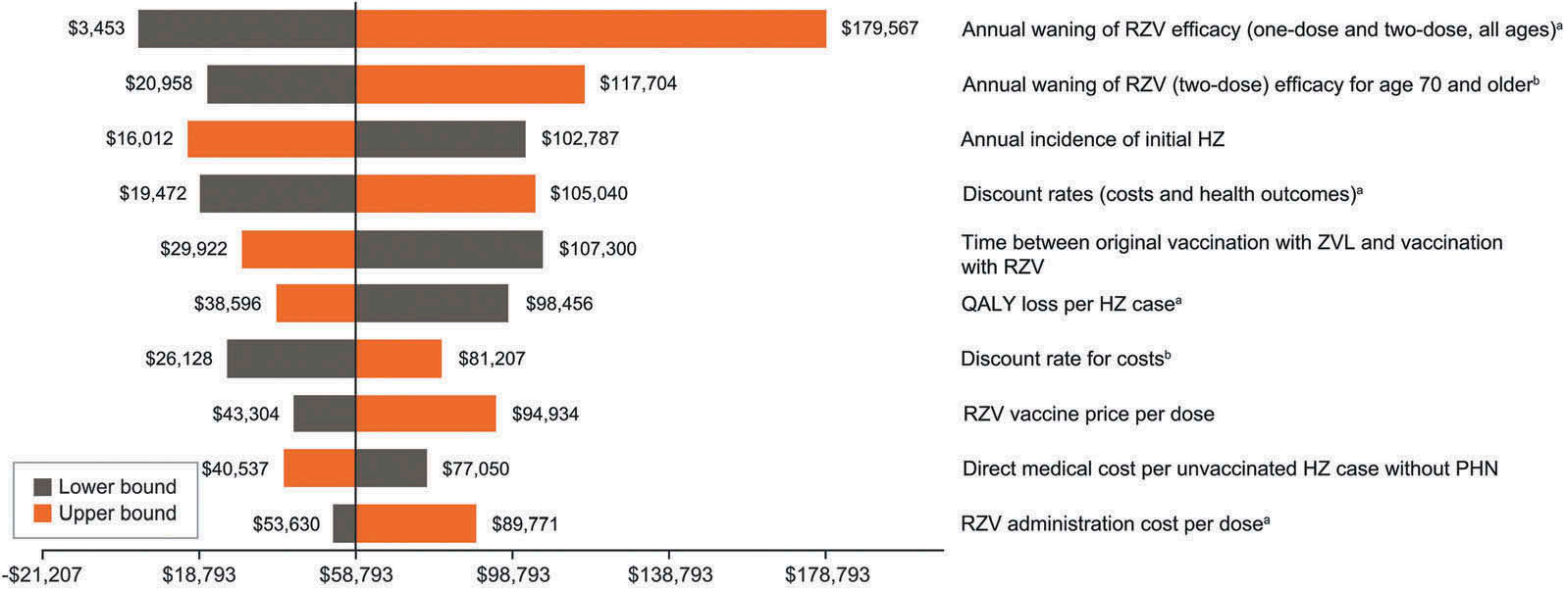
Deterministic sensitivity analysis (DSA) results for ICER of recombinant zoster vaccine (RZV) versus no additional vaccine for US adults aged 60+ year old previously vaccinated against herpes zoster (HZ), top 10 influential variables. The ranges used for the DSA are detailed in Table S2. ^a^Group variation of a set of potentially correlated inputs, each of which is also varied in this DSA individually.^b^Individual variation of an input that is also varied in this DSA grouped with other potentially correlated inputs.

Figure 1 presents the results of the deterministic sensitivity analysis (DSA) for vaccination with RZV versus no additional vaccination. The tornado diagram shows that the incremental cost-effectiveness ratio (ICER) was most sensitive to the following inputs based on their defined ranges: (i) annual waning of RZV efficacy (one-dose and two-dose for all ages pooled), (ii) annual waning of RZV (two-dose) efficacy for adults aged 70+ years old, (iii) annual incidence of initial HZ, (iv) discount rate for costs and health outcomes pooled, and (v) time between original vaccination with ZVL and vaccination with RZV. The highest ICER (or least cost-effective value) was observed when the annual waning of RZV was at its upper bound ($179,567 per QALY gained). The majority (52%) of individual (versus grouped) inputs did not shift the ICER by more than $5,000 in either direction.

**Figure 2.**
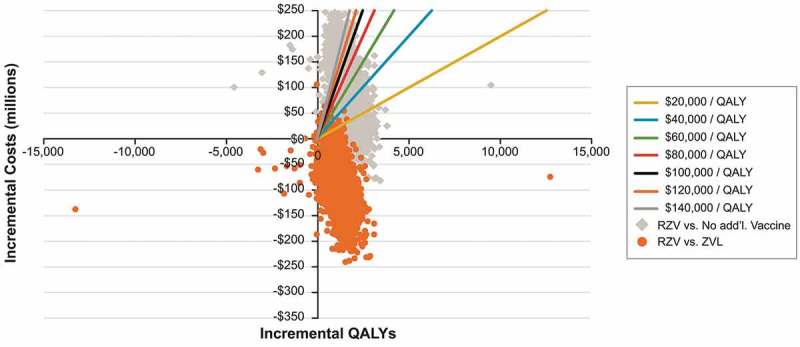
Cost-effectiveness plane showing the incremental costs versus incremental QALYs from 5,000 probabilistic sensitivity analysis simulations for each comparison (recombinant zoster vaccine (RZV) versus no additional vaccination and RZV versus revaccination with zoster vaccine live (ZVL), for US adults aged 60+ years old, previously vaccinated against herpes zoster with ZVL. The ranges used for the PSA are detailed in Table S2.

**Figure 3.**
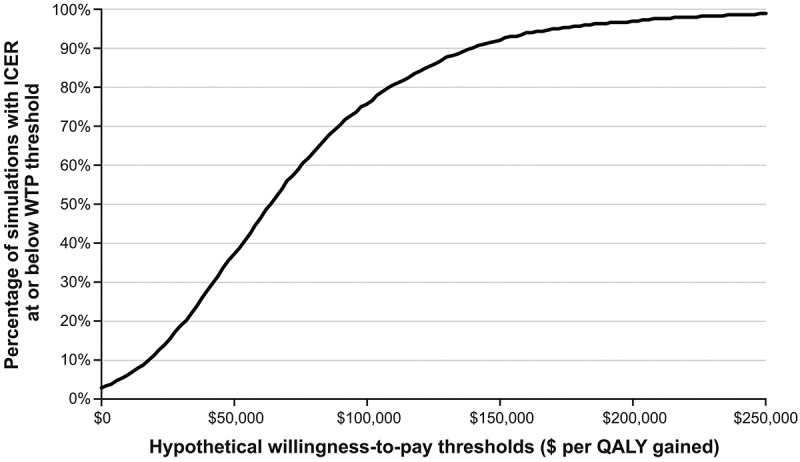
Cost-effectiveness acceptability curve from PSA results for recombinant zoster vaccine (RZV) versus no additional vaccination for US adults aged 60+ years old.

Figure 2 presents the results of the probabilistic sensitivity analysis (PSA) for vaccinating US adults aged 60+ years old previously vaccinated with ZVL, comparing RZV versus no additional vaccination and versus ZVL revaccination. Approximately 75% of simulations comparing vaccinating with RZV versus no additional vaccination resulted in costs per QALY below $100,000, see Figure 3. Approximately 96% of simulations resulted in cost savings when vaccination with RZV replaced ZVL revaccination.

**Figure 4.**
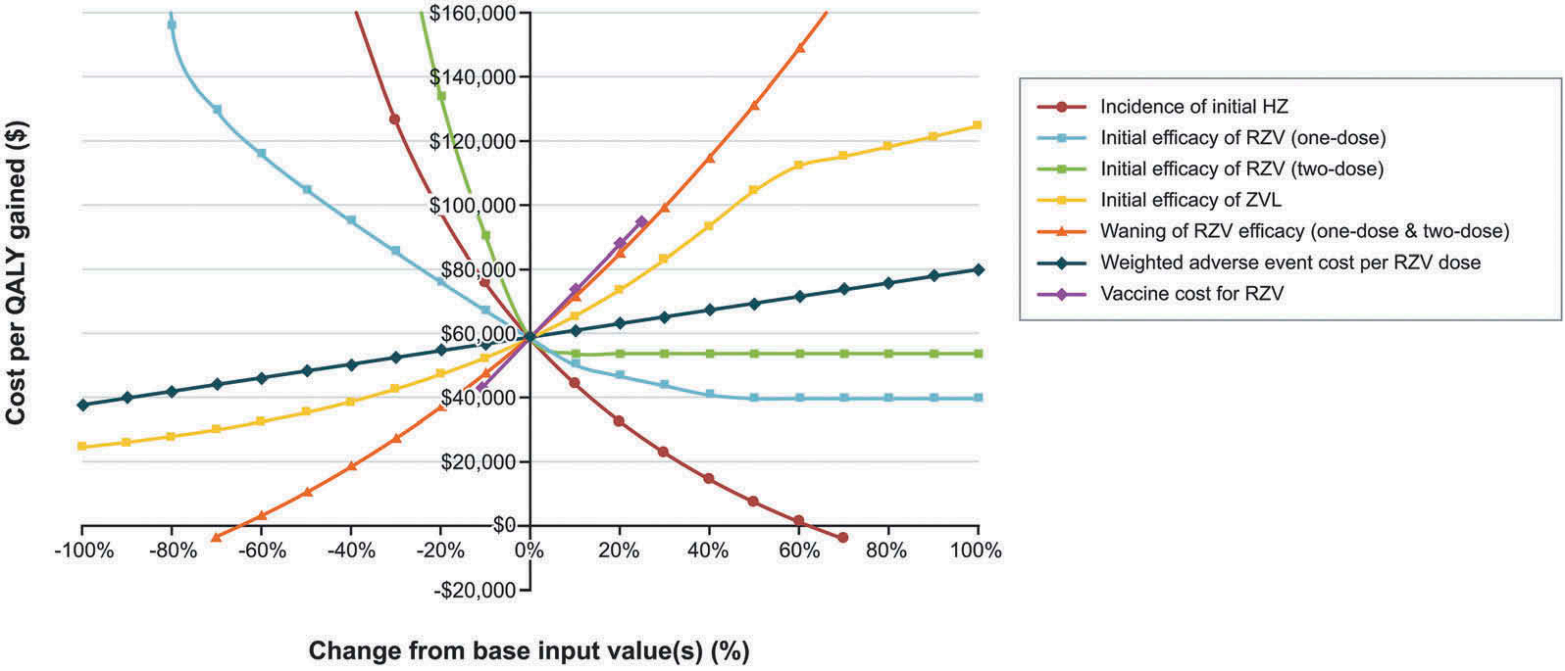
Threshold Analysis: ICER for recombinant zoster vaccine (RZV) versus no additional vaccination for US adults aged 60+ years old previously vaccinated against herpes zoster (HZ) with zoster vaccine live (ZVL) across ranges of values for key inputs. The horizontal lines at various cost-per-QALY values represent different hypothetical willingness-to-pay thresholds.

The results of the threshold analyses for RZV versus no additional vaccination are presented in Figure 4. The model estimated that the initial efficacy of RZV for 2 doses could be roughly 10% lower than the base-case (lower than the lower bound of the 95% confidence interval observed in the clinical trial results) to maintain an ICER for RZV versus no additional vaccination below a threshold of $100,000 per QALY. Similarly, the model estimated that incidence of initial HZ could be approximately 20% lower than base-case estimates and still result in ICERs of less than a threshold of $100,000 per QALY. This analysis also showed that if waning of RZV efficacy remained less than 30% higher than the base-case estimates, the ICER would not exceed $100,000. ICERs were less sensitive to changes in the weighted adverse event (AE) cost per RZV dose from base estimates; as a result, ICERs stayed below a $100,000 threshold until costs were almost 3 times higher than base values.

In the scenario analyses comparing RZV vaccination with no additional vaccination, when we varied the interval between RZV vaccination and the original ZVL vaccination to 1 year and 10 years, ICERs of $107,300 and $32,945 were observed, respectively. Additionally, when effectiveness results of ZVL against PHN as reported by Baxter,^18^ or HZ effectiveness figures as reported by Tseng^19^ were used to model the initial ZVL efficacy and waning parameters, the ICERs were $123,842 and $31,661, respectively (Table S1). Additional vaccination with RZV at age 60 YOA would yield an ICER of $44,962, compared with of $44,789, $73,720, $75,783 at the ages 65, 70 and 80 YOA, respectively.

## Discussion

Since 2008, when ACIP recommended the use of ZVL for the prevention of HZ and its sequelae,^5^ vaccine coverage has increased slowly each year, and by 2016, about 33% of US adults aged 60+ years old reported receipt of the vaccine.^17^ However, as ZVL vaccine efficacy waned, additional vaccination was considered and the cost-effectiveness of ZVL revaccination was studied.^8^ Then following the approval of RZV, ACIP recommended the use of RZV in immunocompetent adults previously vaccinated with ZVL. This is the first post-RZV approval study examining the cost-effectiveness of vaccinating with RZV, compared with either no additional vaccination or ZVL revaccination, basing the analysis on US adults aged 60+ years old who were previously vaccinated with ZVL 5 years earlier. It demonstrated that the RZV vaccine would be cost-effective compared with a no additional vaccination strategy, assuming a hypothetical threshold of $100,000/QALY, and cost-saving compared with ZVL revaccination.

Our conclusions are in line with the model independently developed for the Centers for Disease Control and Prevention (CDC) and presented at the October 2017 ACIP meeting.^12^ The CDC model demonstrated ICERs of less than $60,000 per QALY for all age groups over 60 years of age independent of the time interval between original vaccination with ZVL and vaccination with RZV (i.e. ranging from immediate vaccination (8 weeks after) to 5 years after primary vaccination with ZVL). Our analysis focused on 5 years between vaccinations in the base-case, with an ICER of $58,793, compared with $107,300 and $32,945 when vaccinating 1 or 10 years apart respectively. When determining the optimum time for vaccinating an individual previously vaccinated with ZVL, the person’s age and time since receipt of ZVL should be considered. Since clinical trials of ZVL indicate lower efficacy in older adults, a shorter interval than studied for the population in our analysis may be considered.^17^

Continued protection of the early adopters of HZ vaccination is important for several reasons. As a person ages, their risk of HZ as well as PHN and other complications increases. Additionally, older adults with less physiological reserve and already taking multiple medications for pre-existing chronic conditions may be less able to tolerate HZ and its sequelae.^5^ Healthcare professionals may be slower in prescribing anti-viral therapy if a patient were vaccinated, compounding the quality of life detriment.^20^ Also, to maintain vaccine acceptance, persons should have confidence in a vaccination program.^20^ It is implicit therefore that patients should be protected when they are at greatest risk, pre-empting in the case of HZ, the need for additional vaccination. Healthcare professionals themselves may feel an obligation to offer additional vaccination so as to not misplace the trust patients placed in them to be protected from HZ and its complications when they first received HZ vaccination. The ACIP recommendations allow for this, stating health professionals can “Administer 2 doses of RZV 2–6 months apart to adults who previously received ZVL at least 2 months after ZVL”.^21^

As with every modeling analysis this study has limitations, however with the model design and inputs, intentional decisions were made to minimize unlikely real-world results. The impact of RZV on HZ incidence in the analyses, both in terms of initial protection and maintenance of that protection over time, were based on efficacy data from the RZV ZOE-50 (NCT01165177) and ZOE-70 (NCT01165229) clinical trials^10,11^ and, where those trials could not provide sufficient data, the Shingles Prevention Study clinical trial for ZVL.^1^ The ZOE trials reported efficacy to year 4, therefore because of the lack of long-term data, estimates on waning had to be extrapolated. Although the results were robust to a variety of sensitivity and scenario analyses, real-world RZV effectiveness should be studied further as only in population settings can the long-term duration of protection, adherence to the 2-dose schedule, and the effectiveness and duration of protection in case of non-compliance to the dose series and schedule be elucidated.

Using RZV to vaccinate US adults 60+ years old who were vaccinated 5 years earlier with ZVL, is predicted to be cost-saving compared with ZVL revaccination, and cost-effective when compared with no additional vaccination. Since vaccination with ZVL has been recommended by ACIP for over 10 years in the adults aged 60+ years old, and the effectiveness of vaccination wanes, the aging early adopters of vaccination are becoming increasingly vulnerable to HZ and its complications. This new analysis can be valuable to healthcare professionals, public health decision makers and payers contemplating improving the quality of care of the elderly.

## Methods

### Mathematical model

For the analysis we adapted the previously published ZOster ecoNomic Analysis (ZONA) model^14,22^ to include prior vaccination. The adapted ZONA model is a deterministic multi-cohort Markov model including four hypothetical cohorts split into age groups for people aged 60+ years old (i.e. 60–64, 65–69, 70–79, 80+) with age groupings consistent with those used by the US Census Bureau.^23^ The model, with annual cycle lengths, follows all subjects within a cohort over their remaining lifetimes from the year of vaccination, with all-cause mortality rates taken from the CDC National Center for Health Statistics.^24^ Lifetime outcomes are considered to capture the full effects of HZ vaccination through reduced morbidity. Three different HZ vaccination strategies are compared: no additional vaccination, revaccination with ZVL, and vaccination with RZV. In this analysis all individuals in each cohort have been previously vaccinated against HZ using ZVL, as such they enter the model having residual protection based on initial efficacy of ZVL followed by linear waning. HZ incidence, probability of PHN, and initial efficacy of RZV were modeled using linear rather than step functions to increase consistency between vaccination strategies. Otherwise all input values and assumptions were unchanged.

In the model, vaccinated individuals incur costs for the vaccine price and administration cost. Vaccination reduces the probability of getting HZ and, given a case of HZ, reduces the QALY loss resulting from HZ. Individuals who experience HZ may experience complications. The HZ complications included in the model are PHN as well as ocular, neurological, cutaneous, and other non-pain complications. Recurrent HZ and complications may also occur. Probabilities of moving between health states are age-group–specific and are derived from the US-based peer-reviewed literature, as previously published.^14^ As with the CDC model, presented at ACIP in 2017, our model included direct medical cost input parameters based on a population based study, which reported healthcare utilization and costs due to HZ, PHN and other HZ complications.^12,25^ Indirect costs per HZ case were based on absenteeism and presenteeism-related work loss hours due to HZ based on a telephone survey of HZ patients.^26^ Utilities were calculated from baseline values for individuals without HZ and disutility associated with HZ. Additionally, individuals who are vaccinated have a risk per vaccine dose of experiencing AEs, which result in additional direct costs (healthcare utilization), indirect costs (working hours lost), and QALYs lost. The model includes four possible vaccine-related AEs: local/general reactions, outpatient visits, emergency room visits, and hospitalizations.

### Methodological assumptions

In the base-case analysis we evaluated the ICER, in terms of cost per QALY gained, vaccinating a cohort of 1 million individuals aged 60+ years old in the US population who had been previously vaccinated 5 years prior with ZVL, compared with no additional vaccination. The primary perspective was the societal perspective so as to include both direct medical costs and indirect costs. Costs and outcomes were presented over the remaining lifetimes of individuals. Life years, QALYs, and costs (in US dollars 2016) were discounted at 3% per year, consistent with a lifetime perspective.

### Model inputs

**Figure 5.**
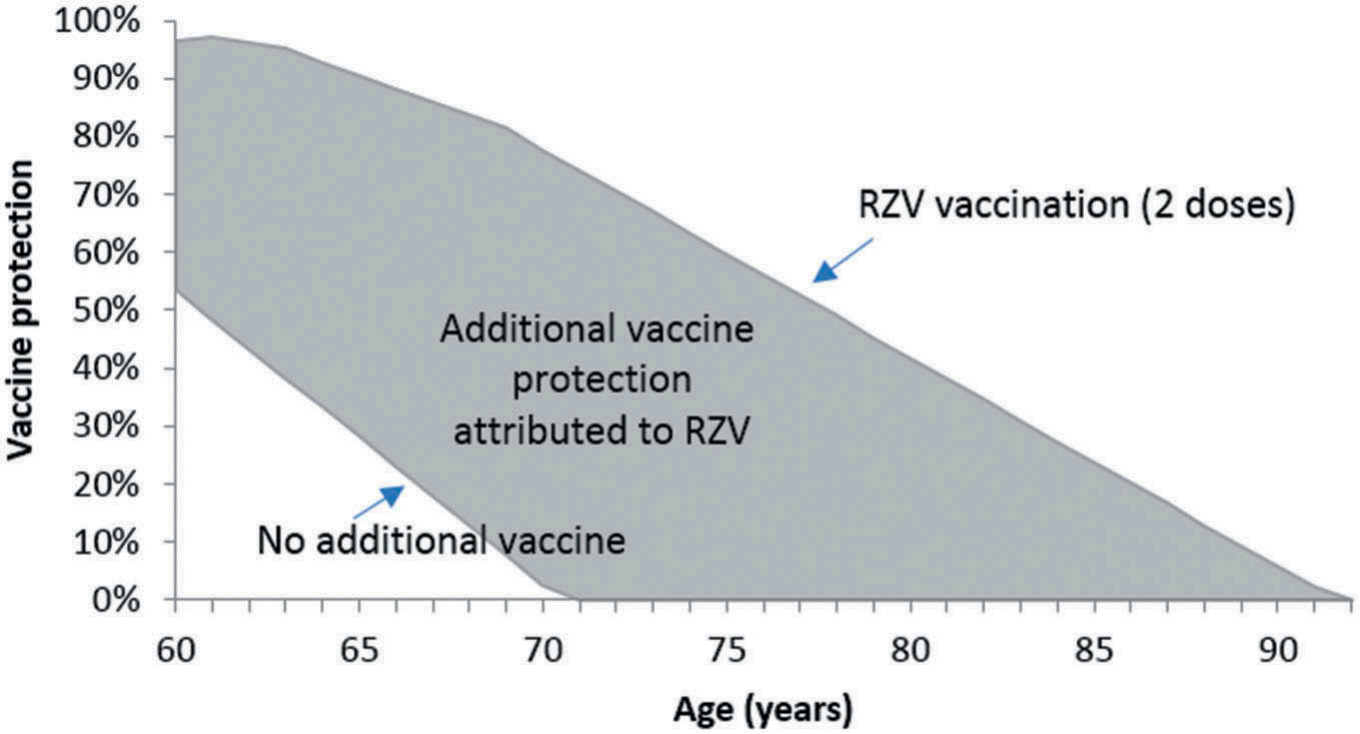
Vaccine efficacy and waning assumptions illustrated for adults vaccinated with 2 doses of recombinant zoster vaccine (RZV) at age 60 years old, following zoster vaccine live (ZVL) 5 years earlier.

Model inputs have been described in detail elsewhere^14,22^ and are summarized in Table S2. Vaccine efficacy inputs for ZVL and RZV were obtained from clinical trials. Additional efficacy of ZVL against PHN cases was included; no additional efficacy of RZV against PHN was included. Figure 5 illustrates the additional effectiveness against HZ attributed to RZV, consistent with data used in the model presented to the CDC.^12^ In the absence of data for one dose of RZV, waning at the same rate as for ZVL was assumed. Vaccination coverage rates were assumed to be 100% for ZVL and RZV first-dose. Compliance with the second dose of RZV (with an interval of 2 months) was 69%, based on the vaccination series completion and compliance rates of hepatitis A and B among US adults.^27^ US-specific (year 2016) HZ-related and vaccine program costs, including management of AEs, were used as previously described.^14^ The base-case RZV price per dose was $140 compared with ZVL $196.91.^28^ Baseline utility values for the US population were taken from Janssen & Szende 2014,^29^ QALY loss per HZ case by vaccination status and PHN status were as reported in Pellissier.^30^

### Sensitivity and scenario analyses

A deterministic sensitivity analysis was conducted with ranges informed by published data where available. The results of the DSA were summarized in a tornado diagram. A probabilistic sensitivity analysis was conducted to consider the impact of the full uncertainty across model inputs on the ICERs for vaccination with RZV (a) versus no additional vaccination, and (b) versus ZVL revaccination. The results from 5,000 Monte Carlo simulations for each analysis, where inputs were simultaneously sampled from probability distributions (gamma for costs and beta for the other varied inputs), were presented on a cost-effectiveness plane, with lines illustrating hypothetical willingness to pay thresholds. The results from the PSA for RZV versus no additional vaccination were also presented as a cost-effectiveness acceptability curve.

Threshold analyses were conducted to investigate, for a selected set of key inputs (HZ incidence; vaccine efficacy and waning; vaccine and AE costs), the values that those inputs could hold and still maintain an ICER for RZV vs. no additional vaccination below various hypothetical willingness-to-pay thresholds ranging from $0 to $160,000 per QALY gained.

Finally, we conducted scenario analyses. Firstly, in place of 5 years between the vaccination with ZVL and RZV, we studied 1 and 10 years. Secondly, we varied ZVL efficacy in line with data from two studies Tseng *et al*. 2016^19^ where the first-year HZ effectiveness of 69% dropped substantially to 4% in year 8, and Baxter *et al*. 2016^18^ where the first-year PHN effectiveness of 83% was better sustained, falling to 70% in year 5; details are included in Table S1.

### Trademark section

*Zostavax* is a trademark of Merck Sharp & Dohme Corp.

*Shingrix* is a trademark of the GSK group of companies.
