# Supplementary material for: Cost-effectiveness of an adjuvanted recombinant zoster vaccine in older adults in the United States who have been previously vaccinated with zoster vaccine live
Source: Hum Vaccin Immunother. 2019 Feb 20;15(4):765–71. doi: 10.1080/21645515.2018.1558689 (PMC6605828; doi:10.1080/21645515.2018.1558689)
Supplement: Supplemental Material [file khvi-15-04-1558689-s001.docx]

Supplementary Online Material

Cost-effectiveness of an adjuvanted Recombinant Zoster Vaccine in older adults in the United States who have been previously vaccinated with Zoster Vaccine Live

Curran D, Patterson B, Van Oorschot D, Buck P, Carrico J, Hicks K, Lee B, Yawn B

Human Vaccines & Immunotherapeutics

Table S1. Scenario analyses results for RZV versus no additional vaccination for US adults aged 60+ years old

| **Scenario** | **ICER** |
| --- | --- |
| **Base** | $58,793 |
| **Scenario 1a:** Shorter time between original vaccination with ZVL and vaccination with RZV (1 year) | $107,300 |
| **Scenario 1b:** Longer time between original vaccination with ZVL and vaccination with RZV (10 years) | $32,945 |
| **Scenario 2a:** Efficacy against HZ and waning of HZ efficacy for ZVL, from Tseng et al. (2016)^1^ | $31,661 |
| **Scenario 2b:** Efficacy against PHN and waning of PHN efficacy for ZVL, from Baxter et al. (2016)^2^ | $123,842 |

Zoster Vaccine Live (ZVL) has been marketed in the US since 2008. As such a maximum of 10 years has occurred since initial vaccination with ZVL. As such we explored additional vaccination with a minimum of 1 year post *Zostavax* vaccination, with a maximum of 10 years, assuming 5 years as the base case.

HZ: herpes zoster; ICER: incremental cost-effectiveness ratio; PHN: post herpetic neuralgia; RZV: recombinant zoster vaccine; US: United States; ZVL: zoster vaccine live.

Table S2. Summary of model inputs ^a^

|  |  | **Range** | |
| --- | --- | --- | --- |
|  | Base-case Value | Lower Bound | Upper Bound |
| **Annual HZ incidence** |  |  |  |
| 60-69, 70-79,  80+ years old | 0.00932, 0.01202, 0.01278 | 0.00746, 0.00962, 0.01022 | 0.01350, 0.01584, 0.01730 |
| Source | ^3^ | Assumed  -20% of base value | ^4^ |
| **Percentage of HZ cases with PHN (%)** |  |  |  |
| 60-69, 70+ years old | 6.20%, 12.70% | 4.96%, 10.16% | 7.44%, 15.24% |
| Source | ^5-7^ | Assumed -20% of base value | Assumed +20% of base value |
| **Annual Recurrent HZ incidence** |  |  |  |
| 60-69, 70-79,  80+ years old | 0.00932, 0.01202, 0.01278 | 0.00110, 0.00143, 0.00143 | 0.01350, 0.01584, 0.01730 |
| Source | ^3,8^ | ^9^ | Assumed Same as annual HZ incidence |
| **Percentage of recurrent HZ cases with PHN (%)** |  |  |  |
| 60-69, 70+ years old | 6.20, 12.70 | 3.72, 7.62 | 8.68, 17.78 |
| Source | ^6-8^ | Assumed Double the range for Initial HZ case PHN percentages | Assumed Double the range for Initial HZ case PHN percentages |
| **Case fatality rate HZ cases (%)** |  |  |  |
| 60-69, 70-79,  80-84, 85+ years old | 0.0022, 0.0062, 0.0240, 0.0734 | 0.0017, 0.0053, 0.0219, 0.0688 | 0.0027, 0.0070, 0.0260, 0.0778 |
| Source | ^10^ | ^10^ |  |
| **Complications (%)** |  |  |  |
| Ocular |  |  |  |
| 60-69, 70-79, 80+ years old | 4.23, 4.53, 6.91 | 1.00, 1.00, 1.00 | 6.57, 6.94, 10.08 |
| Neurological |  |  |  |
| 60-69, 70-79, 80+ years old | 3.17, 5.92, 4.88 | 1.00, 1.00, 1.00 | 5.21, 8.65, 7.57 |
| Cutaneous |  |  |  |
| 60-69, 70-79, 80+ years old | 1.06, 2.09, 2.85 | 0.00, 0.44, 0.77 | 2.25, 3.75, 4.92 |
| Other non-pain |  |  |  |
| 60-69, 70-79, 80+ years old | 1.41, 2.09, 2.85 | 0.04, 0.44, 0.77 | 2.78, 3.75, 4.92 |
| Source | ^11^ | ^11^ | ^11^ |
| **Incidence AEs with RZV** |  |  |  |
| Local/General |  |  |  |
| 60-69, 70+ years old | 0.7980, 0.6880 | ^b^ | ^b^ |
| Outpatient |  |  |  |
| 60-69, 70+ years old | 0.0138, 0.0127 | ^b^ | ^b^ |
| ER |  |  |  |
| 60-69, 70+ years old | 0.0004, 0.0018 | ^b^ | ^b^ |
| Hospitalization |  |  |  |
| All ages | 0.0004 | ^b^ | ^b^ |
| Source for all incidence AEs values with RZV | ^6,7,12^ |  |  |
| **Incidence AEs with ZVL** |  |  |  |
| Local/General |  |  |  |
| 60+ years old | 0.3170 | ^b^ | ^b^ |
| Outpatient |  |  |  |
| All ages | 0.0072 | ^b^ | ^b^ |
| ER |  |  |  |
| All ages | 0.0002 | ^b^ | ^b^ |
| Hospitalization |  |  |  |
| All ages | 0.0003 | ^b^ | ^b^ |
| Source for all incidence AEs values with ZVL | ^12-14^ |  |  |
| **Initial vaccine efficacy against HZ (%)** |  |  |  |
| RZV (One-dose) |  |  |  |
| 60-69, 70+ years old | 90.00, 69.50 | 58.90, 24.90 | 98.90, 89.10 |
| Source | ^15^ | ^15^ | ^15^ |
| RZV (Two-dose) |  |  |  |
| 60-69, 70+ years old | 98.40, 97.84 | 95.00, 94.10 | 100.00, 100.00 |
| Source | ^15^ | ^15^ | ^15^ |
| Zoster Vaccine Live |  |  |  |
| 50-59, 60-69,  70-69, 80+ years old | 69.80, 63.89,  40.85, 18.25 | 54.10, 56.00,  28.00, 0.00 | 80.60, 71.00,  52.00, 48.00 |
| Source | ^16^ | ^16^ | ^16^ |
| **Initial ZVL efficacy against PHN (%)** |  |  |  |
| 50-59, 60-69,  70-69, 80+ years old | 69.80, 65.69,  73.38, 39.51 | 30.8, 25.4,  51.6, 0 | 89.6, 84.2,  85.8, 73.8 |
| Source | ^16^ | Assumed | Assumed |
| **Annual waning of efficacy (%)** |  |  |  |
| RZV (One-dose) |  |  |  |
| Years 1-4, years 5+ | 5.4, 5.1 | 1.0, 3.6 | 7.4, 6.9 |
| Source | ^15^ | ^15^ | ^15^ |
| RZV (Two-dose) |  |  |  |
| ≤70 YOA years 1-4,  ≤70 YOA years 5+, 70+YOA | 1.00,  2.3,  3.6 | 0.0,  0.7,  1.4 | 2.6,  4.6,  6.6 |
| Source | ^15^ | ^15^ | ^15^ |
| Zoster Vaccine Live |  |  |  |
| Years 1-4, years 5+ | 5.4, 5.1 | 4.5, 4.1 | 6.4, 6.0 |
| Source | ^15^ | ^15^ | ^15^ |
| **2nd-dose RZV compliance (%)** | 69.00 | 45.00 | 89.00 |
| Source | ^17^ | ^18^ | ^18^ |
| **Time since primary ZVL vaccination (years)** | 5 | 1 | 11 |
| Source | Assumed | Assumed | Assumed |
| **Scenario vary HZ efficacy** |  |  |  |
| Initial efficacy of ZVL |  |  |  |
| 60-69, 70-79, 80+ years old | 0.69, 0.68, 0.57 |  |  |
| Waning efficacy of ZVL (%) |  |  |  |
| 1-4, 5+ years | 11.6, 6.7 |  |  |
| Source | ^1^ |  |  |
| **Scenario vary PHN efficacy** |  |  |  |
| Initial efficacy of ZVL |  |  |  |
| 60-69, 70-79, 80+ years old | 0.83, 0.83, 0.83 |  |  |
| Waning efficacy of ZVL (%) |  |  |  |
| 1, 2-5, 5+ years | 8.0, 1.0, 5.1 |  |  |
| Source | ^2^ |  |  |
| **RZV cost per dose ($)** | 140.00 | 125.00 | 175.00 |
| Source | ^19^ |  |  |
| **ZVL cost per dose ($)** | 196.91 | 117.12 | 212.67 |
| Source | ^20^ |  |  |
| **Administration cost per dose ($)** | 20.00 | 15.00 | 50.00 |
| Source | ^21^ | ^21^ | ^21^ |
| **Direct costs per HZ case ($)** |  |  |  |
| Without PHN |  |  |  |
| 60-69, 70+ years old | 1,065.48, 1,355.32 | 781.45, 1,092.92 | 1,349.52, 1,617.71 |
| Source | ^22^ | ^22^ | ^22^ |
| With PHN |  |  |  |
| 60-69, 70-79,  80+ years old | 5,274.02, 5,144.53 | 4,219.21, 4,115.62 | 6,328.82, 6,173.44 |
| Source | ^22^ | Assumed -20% of base value | Assumed +20% of base value |
| **Direct cost per complication ($)** |  |  |  |
| Ocular, Neurological,  Cutaneous, Other non-pain | 3,042.17, 7,213.61,  7,214.59, 7,623.38 | 2,433.73, 5,770.89,  5,771.67, 6,098.70 | 3,650.60, 8,656.34, 8,657.50, 9,148.05 |
| Source | ^22^ | Assumed -20% of base value | Assumed +20% of base value |
| **Direct cost per AE ($)** |  |  |  |
| Local/General | 16.48 | ^b^ | ^b^ |
| Source | ^21^ |  |  |
| Outpatient |  |  |  |
| 60-64, 65-69,  70-79, 80+ years old | 361.69, 336.61,  318.30, 314.90 | ^b^ | ^b^ |
| Source | ^23^ |  |  |
| ER |  |  |  |
| 60-64, 65-69,  70-79, 80+ years old | 837.60, 787.43,  750.82, 744.02 | ^b^ | ^b^ |
| Source | ^23^ |  |  |
| Serious (hospitalization) |  |  |  |
| 60-64, 65-69,  70-79, 80+ years old | 7,597.32, 7,346.50, 7,163.44, 7,129.44 | ^b^ | ^b^ |
| Source | ^24,25^ |  |  |
| **Indirect cost due to lost productivity HZ case ($)** |  |  |  |
| 60-64, 65-69,  70-79, 80+ years old | 2,269.10, 1,220.51, 455.19, 313.05 | 1,636.24, 880.11, 328.24, 225.74 | 2,901.96, 1,560.91, 582.15, 400.36 |
| Source | ^26-28^ | Assumed | Assumed |
| **Indirect AE Cost (RZV dose, $)** |  |  |  |
| 60-64, 65-69,  70-79, 80+ years old | 21.52, 21.05,  19.60, 19.53 | 10.76, 10.53,  9.80, 9.77 | 43.04,  42.10, 39.20, 39.06 |
| Source | ^27,28^ | Assumed -50% of base value | Assumed +100% of base value |
| **Indirect AE Cost (ZVL dose, $)** |  |  |  |
| 60-64, 65-69,  70-79, 80+ years old | 10.28, 10.01,  9.82, 9.78 | 5.14, 5.01,  4.91, 4.89 | 20.56, 20.02,  19.64, 19.56 |
| Source | ^27,28^ | Assumed -50% of base value | Assumed +100% of base value |
| **Baseline Utility Values** |  |  |  |
| 60-64, 65-69,  70-79, 80+ years old | 0.8270, 0.8130,  0.7886, 0.7540 | 0.8211, 0.8071,  0.7819, 0.7462 | 0.8329, 0.8189,  0.7952, 0.7618 |
| Source | ^29^ | ^29^ | ^29^ |
| **QALY loss per HZ case without PHN** |  |  |  |
| Unvaccinated |  |  |  |
| 60-69, 70+ years old | 0.010, 0.012 | 0.006, 0.007 | 0.016, 0.018 |
| Vaccinated |  |  |  |
| 60-69, 70+ years old | 0.010, 0.011 | 0.006, 0.007 | 0.014, 0.017 |
| Source | ^30^ | ^30^ | ^30^ |
| **QALY loss per HZ case with PHN** |  |  |  |
| Unvaccinated |  |  |  |
| 60-69, 70+ years old | 0.106, 0.156 | 0.068, 0.100 | 0.162, 0.233 |
| Vaccinated |  |  |  |
| 60-69, 70+ years old | 0.098, 0.091 | 0.063, 0.058 | 0.145, 0.136 |
| Source | ^30^ | ^30^ | ^30^ |
| **QALY loss per AE**  Local/general; Outpatient; ER; Serious | 0.0001; 0.0001;  0.0001; 0.0082 | ^b^ | ^b^ |
| Source | ^10^ | ^10^ | ^10^ |

^a^ For a detailed explanation of all model inputs please see refs. ^12,15^

^b^ The model calculates a weighted AE cost and AE QALY loss per dose based on the incidence of the four AEs and the cost and QALY losses per event. The weighted cost and QALY loss per dose were varied by +100% and –50% in sensitivity analysis

AE: adverse event; ER: emergency room; HZ: herpes zoster; PHN: postherpetic neuralgia; QALY: quality-adjusted life-year; RZV: recombinant zoster vaccine; ZVL: zoster vaccine live.

**Trademark section**

*Zostavax* is a trademark of Merck Sharp & Dohme Corp.

REFERENCES

1. Tseng HF, Harpaz R, Luo Y, Hales CM, Sy LS, Tartof SY, Bialek S, Hechter RC, Jacobsen SJ. Declining Effectiveness of Herpes Zoster Vaccine in Adults Aged ≥60 Years. The Journal of Infectious Diseases. 2016;213(12):1872-5. doi:10.1093/infdis/jiw047

2. Baxter R, Bartlett J, Fireman B, Marks M, Hansen J, Lewis E, Aukes L, Chen Y, Klein NP, Saddier P. Effectiveness of Live Zoster Vaccine in Preventing Postherpetic Neuralgia (PHN). Open Forum Infectious Diseases. 2016;3(suppl_1):128. doi:10.1093/ofid/ofw194.41

3. Johnson BH, Palmer L, Gatwood J, Lenhart G, Kawai K, Acosta CJ. Annual Incidence Rates of Herpes Zoster among an Immunocompetent Population in the United States. BMC Infectious Diseases. 2015;15(1). doi:10.1186/s12879-015-1262-8

4. Tseng HF. Herpes Zoster Vaccine in Older Adults and the Risk of Subsequent Herpes Zoster Disease. Journal American Medical Association. 2011;305(2):160. doi:10.1001/jama.2010.1983

5. Cunningham AL, Lal H, Kovac M, Chlibek R, Hwang S-J, Díez-Domingo J, Godeaux O, Levin MJ, McElhaney JE, Puig-Barberà J, et al. Efficacy of the Herpes Zoster Subunit Vaccine in Adults 70 Years of Age or Older. New England Journal of Medicine. 2016;375(11):1019-32. doi:10.1056/nejmoa1603800

6. GSK. Efficacy, Safety, and Immunogenicity Study of GlaxoSmithKline (GSK) Biologicals’ Herpes Zoster (HZ) Vaccine GSK1437173A in Adults aged ≥ 50 years (ZOSTER-006 study). 2015. [accessed May 30, 2018]. <https://www.gsk-clinicalstudyregister.com/files2/110390%20-%20Clinical-Study-Result-Summary.pdf>.

7. GSK. Efficacy, Safety and Immunogenicity Study of GSK Biologicals’ Herpes Zoster Vaccine GSK1437173A in Adults aged ≥70 years (ZOSTER-022 study). 2016. [accessed May 30, 2018]. <https://www.gsk-clinicalstudyregister.com/files2/113077-Clinical-Study-Result-Summary.pdf>

8. Yawn BP, Wollan PC, Kurland MJ, St. Sauver JL, Saddier P. Herpes Zoster Recurrences More Frequent Than Previously Reported. Mayo Clinic Proceedings. 2011;86(2):88-93. doi:10.4065/mcp.2010.0618

9. Tseng HF, Chi M, Smith N, Marcy SM, Sy LS, Jacobsen SJ. Herpes Zoster Vaccine and the Incidence of Recurrent Herpes Zoster in an Immunocompetent Elderly Population. The Journal of Infectious Diseases. 2012;206(2):190-6. doi:10.1093/infdis/jis334

10. Le P, Rothberg MB. Cost-Effectiveness of Herpes Zoster Vaccine for Persons Aged 50 Years. Annals of Internal Medicine. 2015;163(7):489-97. doi:10.7326/m15-0093

11. Yawn BP, Saddier P, Wollan PC, Sauver JLS, Kurland MJ, Sy LS. A Population-Based Study of the Incidence and Complication Rates of Herpes Zoster Before Zoster Vaccine Introduction. Mayo Clinic Proceedings. 2007;82(11):1341-9. doi:10.4065/82.11.1341

12. Curran D, Patterson B, Varghese L, Van Oorschot D, Buck P, Carrico J, Hicks K, Lee B, Yawn B. Cost-effectiveness of an Adjuvanted Recombinant Zoster Vaccine in older adults in the United States. Vaccine. 2018;36(33):5037-45. doi:10.1016/j.vaccine.2018.07.005

13. Oxman MN. Herpes Zoster Pathogenesis and Cell-Mediated Immunity and Immunosenescence. Journal American Osteopathic Association. 2009;109(6 Suppl 2):S13-7.

14. Schmader KE, Levin MJ, Gnann JW, McNeil SA, Vesikari T, Betts RF, Keay S, Stek JE, Bundick ND, Su SC, et al. Efficacy, Safety, and Tolerability of Herpes Zoster Vaccine in Persons Aged 50-59 Years. Clinical Infectious Diseases. 2012;54(7):922-8. doi:10.1093/cid/cir970

15. Curran D, Van Oorschot D, Varghese L, Oostvogels L, Mrkvan T, Colindres R, von Krempelhuber A, Anastassopoulou A. Assessment of the Potential Public Health Impact of Herpes Zoster Vaccination in Germany. Human Vaccines & Immunotherapeutics. 2017;13(10):2213-21. doi:10.1080/21645515.2017.1345399

16. Merck. Zostavax Prescribing Information. [accessed April 4, 2018]. <https://www.fda.gov/downloads/biologicsbloodvaccines/vaccines/approvedproducts/ucm132831.pdf>.

17. Patterson B, Cheng W, Trofa A, Duchesneau E, Macheca M, Masseria C, Duh M. A Claims-Based Analysis of Hepatitis A, B and A/B Vaccination Series Completion and Compliance among US adults. Value in Health. 2017;20(5):A79.

18. Nelson JC, Bittner RCL, Bounds L, Zhao S, Baggs J, Donahue JG, Hambidge SJ, Jacobsen SJ, Klein NP, Naleway AL, et al. Compliance With Multiple-Dose Vaccine Schedules Among Older Children, Adolescents, and Adults: Results From a Vaccine Safety Datalink Study. American Journal of Public Health. 2009;99(S2):S389-S97. doi:10.2105/ajph.2008.151332

19. Centers for Disease Control and Prevention. CDC Vaccines Price List. 2018. [accessed 2018, May 30]. <https://www.cdc.gov/vaccines/programs/vfc/awardees/vaccine-management/price-list/>

20. Centers for Disease Control and Prevention. CDC Vaccines Price List. 2017. [accessed August 24, 2017]. <https://www.cdc.gov/vaccines/programs/vfc/awardees/vaccine-management/price-list/>

21. Ortega-Sanchez I. Decision and Cost-Effectiveness Analyses of Herpes Zoster Vaccination in Adults 50 Years of Age and Older. [Presentation] Atlanta, GA: US Department of Health and Human Services, CDC. 2013 [accessed 2018, April 4]. https://www.cdc.gov/vaccines/acip/meetings/downloads/slides-2017-10/zoster-03-prosser.pdf

22. Yawn BP, Itzler RF, Wollan PC, Pellissier JM, Sy LS, Saddier P. Health Care Utilization and Cost Burden of Herpes Zoster in a Community Population. Mayo Clinic Proceedings. 2009;84(9):787-94. doi:10.4065/84.9.787

23. Agency for Healthcare Research and Quality (AHRQ). Mean Expenses Per Person with Care for Selected Conditions by Type of Service: United States, 2014. Medical Expenditure Panel Survey Household Component Data. Generated interactively. [accessed February 8, 2017]. <https://meps.ahrq.gov/mepsweb/data_stats/tables_compendia_hh_interactive.jsp?_SERVICE=MEPSSocket0&_PROGRAM=MEPSPGM.TC.SAS&File=HC2Y2014&Table=HC2Y2014_CNDXP_CA&_Debug>=.

24. Le P, Rothberg MB. Determining the Optimal Vaccination Schedule for Herpes Zoster: a Cost-Effectiveness Analysis. Journal of General Internal Medicine. 2017;32(2):159-67. doi:10.1007/s11606-016-3844-6

25. Healthcare Cost and Utilization Project (HCUP). HCUPnet – Hospital Inpatient National Statistics. 2014. [accessed May 30, 2018]. <https://hcupnet.ahrq.gov/#setup>.

26. Singhal PK, Makin C, Pellissier J, Sy L, White R, Saddier P. Work and Productivity Loss Related to Herpes Zoster. Journal of Medical Economics. 2011;14(5):639-45. doi:10.3111/13696998.2011.607482

27. United States Bureau of Labor Statistics. Employment Status of the Civilian Noninstitutional Population by Age, Sex, and Race 2016. [accessed August 24, 2017]. <http://www.bls.gov/cps/cpsaat03.htm>.

28. United States Bureau of Labor Statistics. Median Usual Weekly Earnings of Full-time Wage and Salary Workers by Age, Race, Hispanic or Latino Ethnicity, and Sex, First Quarter 2015 Averages, Not Seasonally Adjusted 2016. [accessed August 24, 2017]. <http://www.bls.gov/news.release/wkyeng.t03.htm>.

29. Szende A, Janssen B. Population Norms for the EQ-5D. In: Szende A, Janssen B, Cabases J, editors. Self-Reported Population Health: An International Perspective based on EQ-5D. Dordrecht: Springer, 2014. p. 19-30.

29. Pellissier JM, Brisson M, Levin MJ. Evaluation of the Cost-Effectiveness in the United States of a Vaccine to Prevent Herpes Zoster and Postherpetic Neuralgia in Older Adults. Vaccine. 2007;25(49):8326-37. doi:10.1016/j.vaccine.2007.09.066
